# Predictive Ability of ^18^F-fluorodeoxyglucose Positron Emission Tomography/computed Tomography for Pathological Complete Response and Prognosis after Neoadjuvant Chemotherapy in Triple-negative Breast Cancer Patients

**DOI:** 10.7508/aojnmb.2016.04.002

**Published:** 2016

**Authors:** Sachiko Kiyoto, Yoshifumi Sugawara, Kohei Hosokawa, Rieko Nishimura, Natsumi Yamashita, Shozo Ohsumi, Teruhito Mochizuki

**Affiliations:** 1 Department of Breast Oncology, National Hospital Organization Shikoku Cancer Center, Matsuyama, Japan; 2 Department of Diagnostic Radiology, National Hospital Organization Shikoku Cancer Center, Matsuyama, Japan; 3 Department of Clinical Laboratory, National Hospital Organization Shikoku Cancer Center, Matsuyama, Japan; 4 Section of Cancer Prevention and Epidemiology, Clinical Research Center, National Hospital Organization Shikoku Cancer Center, Matsuyama, Japan; 5 Department of Radiology, Ehime University, Matsuyama, Japan

**Keywords:** FDG-PET/CT, Neoadjuvant Chemotherapy, Metabolic Response, Prognosis, Triple negative breast cancer

## Abstract

**Objective(s):**

The mortality of patients with locally advanced triple-negative breast cancer (TNBC) is high, and pathological complete response (pCR) to neoadjuvant chemotherapy (NAC) is associated with improved prognosis. This retrospective study was designed and powered to investigate the ability of ^18^F-fluorodeoxyglucose positron emission tomography/computed tomography (^18^F-FDG-PET/CT) to predict pathological response to NAC and prognosis after NAC.

**Methods:**

The data of 32 consecutive women with clinical stage II or III TNBC from January 2006 to December 2013 in our institution who underwent FDG-PET/CT at baseline and after NAC were retrospectively analyzed. The maximum standardized uptake value (SUV_max_) in the primary tumor at each examination and the change in SUV_max_ (ΔSUV_max_) between the two scans were measured. Correlations between PET parameters and pathological response, and correlations between PET parameters and disease-free survival (DFS) were examined.

**Results:**

At the completion of NAC, surgery showed pCR in 7 patients, while 25 had residual tumor, so-called non-pCR. Median follow-up was 39.0 months. Of the non-pCR patients, 9 relapsed at 3 years. Of all assessed clinical, biological, and PET parameters, N-stage, clinical stage, and ΔSUV_max_ were predictors of pathological response (*p* value of 0.0288, 0.0068, 0.0068 respectively; Fischer’s exact test). The cut-off value of ΔSUV_max_ to differentiate pCR evaluated by the receiver operating characteristic (ROC) curve analysis was 81.3%. Three-year disease-free survival (DFS) was lower in patients with non-pCR than in patients with pCR (*p*=0.328, log-rank test). The cut-off value of ΔSUV_max_ to differentiate 3-year DFS evaluated by the ROC analysis was 15.9%. In all cases, 3-year DFS was lower in patients with ΔSUV_max_ <15.9% than in patients with ΔSUV_max_ ≥15.9% (*P*=0.0078, log-rank test). In non-pCR patients, 3-year DFS was lower in patients with ΔSUV_max_ <15.9% than in patients with ΔSUV_max_ ≥15.9% (*P*=0.0238, log-rank test).

**Conclusion:**

FDG-PET/CT at baseline and after NAC could predict pathological response to NAC before surgery and the clinical outcome after surgery in locally advanced TNBC patients.

## Introduction

Most locally advanced breast cancers are currently treated with neoadjuvant chemotherapy (NAC) followed by breast and axillary surgery. Triple-negative breast cancer (TNBC), characterized by lack of estrogen receptor (ER) and progesterone receptor (PR) and absence of human epidermal growth factor receptor type 2 (HER2) over-expression, accounts for 10-20% of invasive breast cancers (1, 2). Patients with TNBC have a relatively poor outcome, with higher rates of early relapse than other types of breast tumors. However, these aggressive tumors have more intrinsic responsiveness to NAC than ER-positive tumors. Furthermore, TNBC patients with pathological complete response (pCR) after NAC have a good prognosis, while the prognosis is particularly poor in patients who do not achieve a pCR (3, 4). Therefore, achieving pCR for TNBC patients is a very important clinical objective.

For patients with large or locally advanced breast cancer, positron emission tomography/computed tomography (PET/CT) with ^18^F-fluorodeoxyglucose (^18^F-FDG) is gaining importance for staging (5, 6), and the early changes in PET parameters in the primary breast tumor can serve as a potential predictive biomarker of response to NAC (7, 8). However, performing FDG-PET/CT before and after NAC is still not common at present. The present retrospective study investigated the ability of PET parameters to predict pathological response and prognosis in a series of TNBC patients. The predictive value of PET was also compared to that of baseline clinical or biological factors.

## Materials and Methods

### Patients

There were 53 consecutive patients with clinical stage II or III breast carcinoma and triple-negative phenotype defined by core needle biopsy before surgery from January 2006 to December 2013 in our institution. The inclusion criteria for the retrospective review were that NAC was performed before surgery and that FDG-PET/CT was performed both before and after NAC; 34 patients met the criteria. The exclusion criteria for the retrospective review were: metastatic breast cancer (M1) (1 patient); inflammatory breast cancer (no patients); synchronous ipsilateral multiple breast cancer (no patients); synchronous and metachronous bilateral breast cancers (no patients); synchronous and metachronous multiple cancers (1 patient); and unknown incomplete follow-up (no patients). Finally, 32 consecutive patients were analyzed retrospectively. Our institutional review board approved this study and waived the need for informed consent on the basis of the retrospective design.

### Histological diagnosis and receptor status of the tumor

Core needle biopsy specimens before NAC were used for histological diagnosis. The National Surgical Adjuvant Study (N-SAS) grading for invasive ductal carcinoma was used for histological grading.

Tumors were defined as triple-negative on the basis of the results of immunohistochemical (IHC) staining performed on formalin-fixed, paraffin-embedded tissue, using an automated immunostainer (Ventana BenchMark ULTRA, Roche Diagnostics, Basel, Switzerland). Receptor status was determined at the invasive area of the tumor. Hormone receptor status of the tumor was considered positive if ≥1% of tumor cells showed positive nuclear staining. HER2 status was considered over-expressed if uniform and intense membranous staining was seen in >30% of tumor cells on IHC (IHC 3+). An equivocal result (IHC 2+) was further tested by fluorescent in situ hybridization (FISH).

### Neoadjuvant chemotherapy

Sixteen patients received FEC-DTX (4 cycles of fluorouracil 500 mg/m^2^ plus epirubicin 100 mg/m^2^ plus cyclophosphamide 500 mg/m^2^ administered every 3 weeks, followed by 4 cycles of docetaxel 75 mg/m^2^ administered every 3 weeks). Four patients received FEC-PTX (4 cycles of fluorouracil 500 mg/m^2^ plus epirubicin 100 mg/m^2^ plus cyclophosphamide 500 mg/m^2^ administered every 3 weeks, followed by 12 cycles of paclitaxel 80 mg/m^2^ administered every week). Three patients received AC-DTX (4 cycles of adriamycin 60 mg/m^2^ plus cyclophosphamide 600 mg/m^2^ administered every 3 weeks, followed by 4 cycles of docetaxel 75 mg/m^2^ administered every 3 weeks). Four patients received AC-PTX (4 cycles of adriamycin 60 mg/m^2^ plus cyclophosphamide 600 mg/m^2^ administered every 3 weeks, followed by 12 cycles of paclitaxel 80 mg/m^2^ administered every week). Five patients received other chemotherapy regimens (PTX/CBDCA-FEC, EC-PTX, TAC, AC, PTX; one each).

### FDG-PET/CT imaging

Details of the scanning were previously reported by Nakajima et al. (9). In brief, patients fasted for 4 hours before the intravenous injection of approximately 3.0 MBq/kg body weight of ^18^F-FDG. The serum glucose level immediately before the injection was measured to ensure that it was less than 120 mg/dl. Dual-modality PET-CT imaging was performed using an Aquiduo (Toshiba Medical Systems Corporation, Otawara, Japan). Whole-body CT covered a region ranging from the head to the upper thighs. Whole-body PET images with attenuation correction were acquired about 90 min later. The acquisition time of PET was adapted according to the patients’ weight. PET images were scatter-corrected and iteratively reconstructed into a 128×128 matrix with 1.34 zooming, using interactive algorithms (ordered-subset expectation maximization, 2 iterations, 14 subsets) and the CT-based attenuation map.

The PET/CT data were transferred to a Vox-Base II workstation (J-MAC Systems, Inc., Sapporo, Japan). The images of CT, PET and fused PET/CT were separately displayed on an image viewer. PET images were displayed with SUV of 0-6.

A 3D region of interest (3D-ROI) was manually placed over an area of activity on the primary tumor in attenuated corrected images, and SUV_max_ (maximum SUV value) in the 3D-ROI was automatically obtained. The change in SUV_max_ after NAC was defined as follows: ΔSUV_max_(%)=100×(baseline SUV_max_ - after NAC SUV_max_)/baseline SUV_max_).

### Pathological assessment after neoadjuvant chemotherapy

The tumor site of surgically resected specimens was cut into serial strips with width of 1 cm, and the whole cut surface was examined histologically. Pathological complete response (pCR) was defined as no evidence of residual invasive or non-invasive carcinoma in the breast tissue and lymph nodes (ypT0/ypN0).

### Statistical analysis

Correlations between pathologic response and SUV parameters (SUV_max_ at baseline and after NAC, ΔSUV_max_) were examined with Wilcoxon rank-sum tests. The predictive performance for the identification of pCR and relapse were evaluated using receiver operating characteristic (ROC) curve analysis.

Associations between baseline clinical and biological parameters (tumor size, axillary status, etc.) and pathological response were examined with Fisher’s exact tests and multivariate exact logistic regression.

The log-rank test was used to examine the associations between PET parameters and disease-free survival (DFS), and between baseline clinical and biological factors and DFS. Survival curves were drawn using the Kaplan-Meier method.

Statistical analyses were performed using JMP software (version 11) and Stata 11. All tests were two-sided, and *P* values <0.05 were considered significant.

## Results

Baseline patient and tumor characteristics of the 32 TNBC patients are summarized in Table 1.

**Table 1:** Characteristics of the 32 triple-negative breast cancer (TNBC) patients

	No. of patients (%)
Age at surgery (y), median (range)	54 (31-71)
Follow-up after surgery, months, median (range)	39.0 (5.8-91.2)
Clinical tumor classification^a^	
T1	1 (3.1)
T2	22 (68.8)
T3	5 (15.6)
T4	4 (12.5)
Clinical lymph node classification^a^	
N0	7 (21.9)
N1	13 (40.6)
N2	4 (12.5)
N3	8 (25.0)
AJCC Clinical stage^a^	
IIA	5 (15.6)
IIB	11 (34.4)
IIIA	7 (21.9)
IIIB	1 (3.1)
IIIC	8 (25.0)
Histological type	
Invasive ductal carcinoma	30 (93.8)
Metaplastic carcinoma	1 (3.1)
Secretory carcinoma	1 (3.1)
Nuclear grade	
Grade 1	5 (15.6)
Grade 2	12 (37.5)
Grade 3	15 (46.9)
Chemotherapy regimen	
FEC-DTX	16 (50.0)
FEC-PTX	4 (12.5)
AC-DTX	3 (9.4)
AC-PTX	4 (12.5)
Others	5 (15.6)
Completion of NAC	
Complete	30 (93.8)
Incomplete	2 (6.2)
Surgery: breast	
Mastectomy (Bt)	16 (50.0)
Breast-conserving therapy (Bp)	16 (50.0)
Surgery: lymph nodes	
Sentinel lymph node biopsy (SN)	3 (9.4)
Axillary dissection level I	15 (46.8)
Axillary dissection level II	4 (12.5)
Axillary dissection level III	10 (31.3)
Pathologic response	
pCR(T0N0)	7 (21.9)
Non-pCR	25 (78.1)
Relapse	
No relapse	20 (62.5)
Local or regional relapse only	2 (6.2)
Distant relapse	10 (31.3)

FEC-DTX, sequential regimen of four cycles of fluorouracil 500 mg/m^2^ plus epirubicin 100 mg/m^2^ plus cyclophosphamide 500 mg/m^2^ administered every 3 weeks, followed by four cycles of docetaxel 75 mg/m^2^ administered every 3 weeks; FEC-PTX, sequential regimen of four cycles of fluorouracil 500 mg/m^2^ plus epirubicin 100 mg/m^2^ plus cyclophosphamide 500 mg/m^2^ administered every 3 weeks, followed by 12 cycles of paclitaxel 80 mg/m^2^ administered every week; AC-DTX, sequential regimen of four cycles of adriamycin 60 mg/m^2^ plus cyclophosphamide 600 mg/m^2^ administered every 3 weeks, followed by four cycles of docetaxel 75 mg/m^2^ administered every 3 weeks; AC-PTX, sequential regimen of four cycles of adriamycin 60 mg/m^2^ plus cyclophosphamide 600 mg/m^2^ administered every 3 weeks, followed by 12 cycles of paclitaxel 80 mg/m^2^ administered every week.

a Clinical stage before ^18^F-fluorodeoxyglucose positron emission tomography/computed tomography (^18^F-FDG-PET/CT) according to the seventh edition of the American Joint Committee on Cancer (AJCC) staging manual.

At completion of NAC, breast-conserving surgery was performed in 16 women, and mastectomy was performed in 16. Sentinel node biopsy was performed in 3 women, and axillary lymph node dissection was performed in 29 women. Histopathology showed pCR in 7 patients (21.9%) and non-pCR in 25 (78.1%).

### Association between pCR and DFS

**Figure 1:**
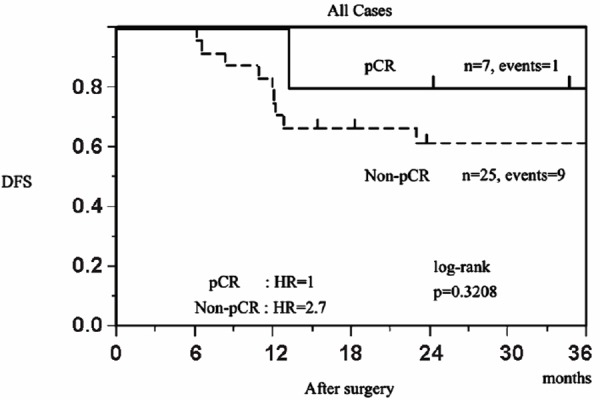
Kaplan-Meier disease-free survival (DFS) curves by pathological response at surgery after neoadjuvant chemotherapy

Median follow-up was 39.0 months (range 5.8-91.2 months). The 3-year DFS was 66.0% (95% confidence interval (CI), 47.4-80.7%). Twelve patients relapsed, of whom seven died. Ten relapses occurred during the first 36 months of follow-up, of which nine occurred in the group of patients with non-pCR, whereas only one occurred in the group of patients with pCR (log-rank test; *P*=0.3208). The 3-year DFS was 85.7% (30.9-97.3%) in patients with pCR versus 64.0% (41.4-78.9%) in those with non-pCR (Figure 1).

### PET parameters and pathological response (Table 2)

**Table 2:** Associations between positron emission tomography (PET) parameters and pathological response at completion of NAC

PET parameter	Median (min/max)			*p* ^a^

Primary tumor	All patients (n=32)	pCR (n=7)	Non-pCR (n=25)	
SUV_max_ baseline	9.95(2.7/31.8)	7.5(2.7/31.8)	10.0(3.5/29.2)	0.6485
SUV_max_ after NAC	1.55(0.5/18.0)	1.0(0.5/1.2)	2.6(0.6/18.0)	0.0040
ΔSUV_max_	80.5%(-13.6/96.9)	87.7%(81.3/96.9)	75.2%(-13.6/94.7)	0.0201

a Difference between pathological complete response (pCR) and non-pCR rates with the Wilcoxon rank-sum test. Bold numbers correspond to significant p values.

At baseline, SUV_max_ of breast tumor ranged between 2.7 and 31.8 (median=9.95). There was no correlation between baseline SUV_max_ of the primary tumor and pathological response (median SUV_max_=7.5 (range 2.7-31.8) in the pCR group versus 10.0 (range 3.5-29.2) in the non-pCR group; *P*=0.6485). SUV_max_ after NAC and ΔSUV_max_ of breast tumor ranged between 0.5-18.0 (median=1.55) and 13.6-96.9% (median=80.5%), respectively. There were strong correlations between SUV_max_ after NAC of the primary tumor and pathological response (median SUV_max_=1.0 (range 0.5-1.2) in the pCR group versus 2.6 (range 0.8-18.0) in the non-pCR group; *P*=0.0040) and between ΔSUV_max_ of the primary tumor and pathological response (median ΔSUV_max_=87.7% (range 81.3-96.9%) in the pCR group versus 75.2% (range: 13.6-94.7%) in the non-pCR group; *P*=0.0201).

### The choice of the ΔSUV_max_ threshold to define metabolic response

**Figure 2:**
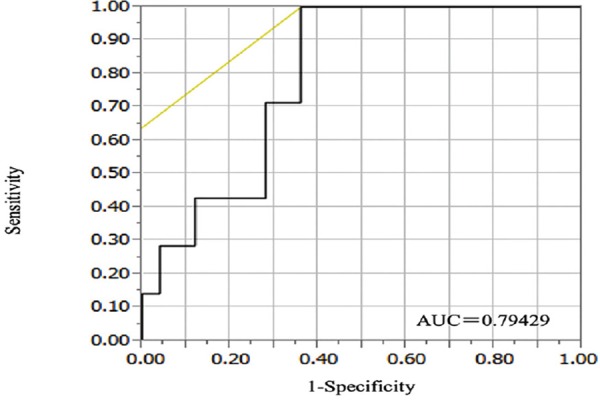
Receiver operating characteristic (ROC) curve of ΔSUV_max_ for the identification of pCR after surgery

A cut-off of 81.3% for ΔSUV_max_ in the primary tumor offered the best accuracy in predicting pCR (AUC=0.79429, accuracy=71.9%; positive predictive value (PPV) =77.8% and negative predictive value (NPV) =100%) (Figure 2). The 81.3% cut-off was selected to define metabolic response. With this cut-off, there were 16 good metabolic responders (ΔSUV_max_≥81.3%) and 16 poor responders (ΔSUV_max_<81.3%). The pCR rates in these group were 43.8% and 0% (*P*=0.0068), respectively. Pathological CR was predicted with a PPV of 77.8%, NPV of 100%, and accuracy of 71.9%. The very high NPV means that poor response (ΔSUV_max_<81.3%) on PET/CT always indicates non-pCR.

### Relationship between ΔSUV_max_ and DFS

The threshold ΔSUV_max_ of 81.3% was not able to predict relapse. The 3-year DFS rate was 31.3% in metabolic responders (ΔSUV_max_≥81.3%) versus 31.3% in poor responders (<81.3%; *P*=1.000).

**Figure 3:**
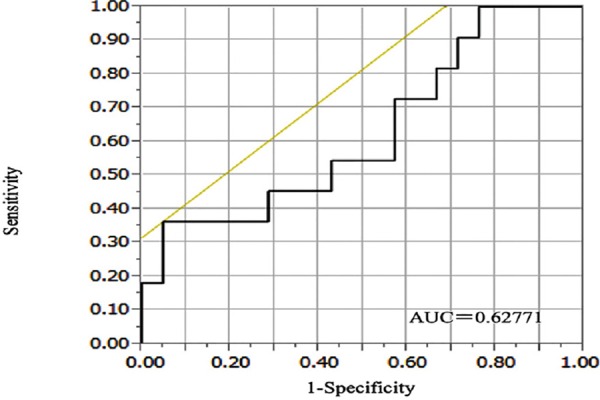
Receiver operating characteristic (ROC) curve of ΔSUV_max_ for the identification of relapse at 3 years after surgery

**Figure 4:**
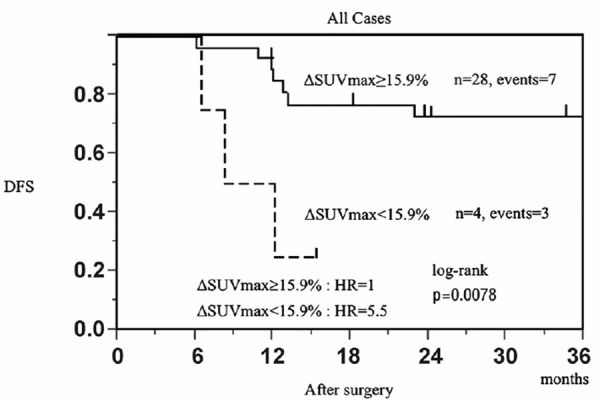
Kaplan-Meier distant disease-free survival (DFS) curves according to the decrease of the maximum standardized uptake value (SUV_max_) in the primary tumor after neoadjuvant chemotherapy

However, the cut-off ΔSUV_max_ of 15.9% in the primary tumor offered the best accuracy in predicting relapse (AUC=0.6277, accuracy=71.9%, PPV=71.4%, and NPV=75.0%) (Figure 3). 3-year DFS was 75% (52.4-86.4%) for metabolic responders and 25.0% (3.4-76.2%) for non-responders. Figure 4 shows the Kaplan-Meier DFS curves obtained when using a cut-off value of 15.9% to differentiate metabolic responders (≥15.9% decrease in SUV_max_ in the tumor) from non-responders. The hazard ratio (HR) of relapse was 5.51 (95% CI =1.14-21.4) for patients with ΔSUV_max_<15.9% after NAC in comparison to those with ΔSUV_max_ ≥15.9% (*P*=0.0078, log-rank test).

**Figure 5:**
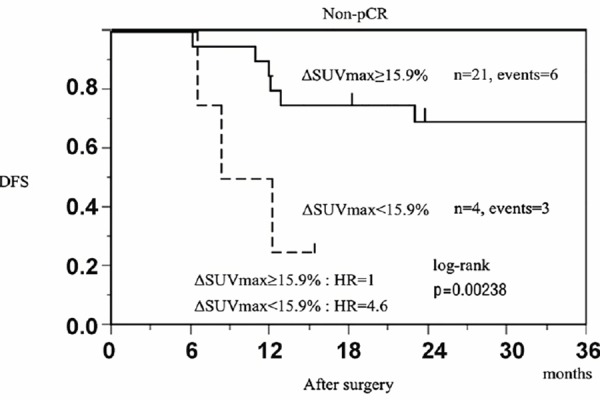
Kaplan-Meier distant disease-free survival (DFS) curves according to the decrease of the maximum standardized uptake value (SUV_max_) in the primary tumor after neoadjuvant chemotherapy in non-pCR patients

Furthermore, in non-pCR patients, with the ΔSUV_max_ in the primary tumor cut-off of 15.9%, the Kaplan-Meier DFS curves showed that the HR of relapse was 4.61 (95% CI=0.93-19.20) for patients with ΔSUV_max_<15.9% after NAC compared to those with ΔSUV_max_ ≥15.9% (*P*=0.0024, log-rank test) (Figure 5).

### Predictions with baseline clinical and biological parameters

Pathologic response was not associated with clinical tumor status at presentation (T1/2 versus T3/4; *P*=0.1492), with histological type (ductal versus others; *P*=1.0000), or with tumor grade (grade 1 versus grade 2/3; *P*=0.5603) (Table 3).

**Table 3:** Associations between clinical variables, histological variables, and ΔSUV_max_ with response at NAC completion

	N	pCR (n=7)	non-pCR(n=25)	*p*^a^
T-stage^b^				
T1-T2	22	7	15	0.1492
T3-T4	10	0	10	
N-stage^b^				
N0-N1	20	7	13	0.0288
N2-N3	12	0	12	
Clinical stage^b^				
II	16	7	9	0.0068
III	16	0	16	
Histological type				
Ductal	30	7	23	1.0000
Others	2	0	2	
Tumor grade				
1	5	0	5	0.5603
2,3	27	7	20	
ΔSUV_max_ (cut off 81.3%)				
<81.3%	16	0	16	0.0068
≥81.3%	16	7	9	

a Difference between pathological complete response (pCR) and non-pCR rates with Fisher’s exact tests. Bold numbers correspond to significant p values.

b **Clinical stage before** FDG-PET/CT according to the seventh edition of the AJCC staging manual.

Pathologic complete response was more frequent for N0/1 than N2/3 (35% versus 0%, *P*=0.0288) and for clinical stage II than III (43.8% versus 0%, *P*=0.0068). The overall accuracy predicting the pathological outcome was 59.4% for N-stage and 71.9% for clinical stage.

Neither baseline N-Stage nor clinical stage was associated with relapse. The HR was 2.10 (95% CI=0.58-7.55) for N0/1 compared to N02/3 tumors (*P*=0.23, log-rank test). The HR was 1.06 (95% CI=0.29-3.82) for clinical stage II compared to stage III tumors (*P*=0.93, log-rank test).

### Multivariate analysis

The results of multivariate exact logistic regression evaluating PET parameters and pathological response at completion of NAC are presented in Table 4. It was found that ΔSUV_max_ ≥81.3% was significantly predictive of pCR with adjustment for clinical stage II (odds ratio 20.27; *P*=0.0063 versus 20.27; *P*=0.0063) and N-stage 0-1 (odds ratio 13.11; *P*=0.0210 versus 22.20; *P*=0.0031) (Table 4).

**Table 4:** Multivariate exact logistic regression evaluating parameters and pathological response at NAC completion

Parameter	Odds ratio for pCR	95%CI	*p*^a^
Clinical stage (II vs. III)	20.27	(2.18 - +Inf)	0.0063
ΔSUV_max_ (≥81.3% vs. <81.3%)	20.27	(2.18 - +Inf)	0.0063

Parameter	Odds ratio for pCR	95%CI	

N-stage (N0-N1 vs. III)	13.11	(1.43 - +Inf)	0.0210
ΔSUV_max_ (≥81.3% vs. <81.3%)	20.27	(2.58 - +Inf)	0.0031

a Difference between clinical pathological complete response (pCR) and non-pCR rates with exact logistic regression. Bold numbers correspond to significant p values.

## Discussion

Pathological complete response is a surrogate maker when TNBC patients are treated by NAC (3, 4). In this retrospective study of 32 women, the overall pCR rate was 21.9%, and the 3-year DFS was 85.7% (30.9-97.3%) in patients with pCR versus 64.0% (41.4-78.9%) in those with non-pCR at surgery. The use of baseline FDG-PET/CT staging could have contributed by excluding patients with occult distant metastases (6).

Regarding clinical and biological parameters, pCR was more frequent for N0/1 tumors than for N2/3 tumors and for clinical stage II compared to stage III, which is in agreement with other reports (10). The pCR rate in the present series was 25.9% (7/27) in patients with high-grade (grade 2/3) invasive ductal carcinoma (IDC), which was the main subtype, while the rate was very low in patients with other tumor types (invasive lobular carcinoma and special type) (0/2). Nagao et al. reported, in a group of 562 patients with breast carcinoma, that the response of metaplastic carcinoma was also significantly poorer to NAC than to IDC (*P*=0.003), and about 50% of patients with metaplastic carcinoma developed progressive disease, which was significantly higher than the recurrence rate in those with IDC (*P*<0.001) (11). With regard to tumor grade, in the meta-analysis by Cortazar et al. (12), the pCR rate in patients with breast cancer (mixed phenotypes) was higher among the 3,217 with grade 3 than among the 4,392 with grade 2 tumors (25.8% vs. 12.3%). One explanation could be that high-grade tumors are more proliferative and more sensitive to chemotherapy than lower grade tumors. However, the prognosis in patients with grade 3 tumors who do not achieve pCR is poor. In the present series, no patients with grade 1 tumors achieved pCR (recurrence rate=3/5, 60%); among grade 2/3 tumors, the recurrence rate was substantially higher with non-pCR (6/20, 30%) than with pCR (1/7, 14.3%).

PET after NAC was a significant predictor of pathological outcome, and the decrease in FDG uptake (ΔSUV_max_) on PET after NAC was a good predictor of pCR (Table 2). The median ΔSUV_max_ measured in the primary tumor was 87.7% in patients who achieved pCR versus 75.2% in patients who did not (*P*=0.02) (Table 2). Results from the present study showed that a cut-off of a 15.9% decrease in SUV_max_ of the primary tumor offers a high accuracy in predicting DFS/relapse. The 3-year DFS was 75.0% (52.4-86.4%) in metabolic responders versus 25.0% (3.4-76.2%) in non-responders (*P*=0.0078, log-rank test).

A cut-off of an 81.3% decrease in SUV_max_ offered the best accuracy in predicting pathological response. Pathological CR was identified with a sensitivity of 100%, specificity of 64.0%, PPV of 77.8%, and NPV of 100%. However, a cut-off of 15.9% offered the best accuracy in predicting DFS. All 7 patients who achieved pCR were well classified, but it was not significant (*P*=0.5523). When using FDG-PET/CT at baseline and after NAC as a surrogate marker for poor response to NAC, an effective cut-off is needed to recommend closer follow-up after surgery to detect relapse early, especially for TNBC patients (10, 13).

Our study had some limitations. This was a retrospective study, it included a small number of patients, and the chemotherapy regimens for NAC were not uniform in all patients.

Only the response of the SUV_max_ of the primary tumor was evaluated, but some previous studies evaluated the response in the primary tumor and axillary lymph nodes (10, 14, 18). In general, NAC was performed based on the nature of the primary tumor, not on the status of the axillary lymph nodes. Lymph node biopsy was not mandatory in the NCCN Clinical Practice Guidelines in Oncology, breast cancer Version 3, 2015. Therefore, in this study, the nature and SUV_max_ of FDG of the primary tumor were used, not of the lymph nodes. It has been shown that analysis including the axillary lymph nodes would not improve the results for predicting pCR over breast tumor alone in triple-negative breast cancer by Groheux et al. (10, 18).

In the present study, FDG-PET/CT was performed at baseline and after NAC, but there was no interim FDG-PET/CT. Several previous reports showed the ability of interim PET after one or two cycles of NAC in TNBC patients to predict pathological response and the outcome soon after surgery (10, 14). However, today, in our country, the clinical relevance of PET for everyday practice is still limited because its use is restricted by the medical insurance system. There is no insurance coverage for frequent PET; for example, PET after two cycles of chemotherapy in regular treatment has not been approved in our country. At present, the second PET could be performed only after chemotherapy for re-staging before surgery. In this study, PET could not predict the effectiveness of chemotherapy early, but it could predict pCR with a high negative predictive value (100%) even after completion of chemotherapy, and it could predict the outcome after surgery by the changes in SUV_max_ from baseline to accomplishment of chemotherapy. This result may be meaningful in areas that have insurance coverage for PET that is similar to that in Japan.

Some reports have shown that the same cut-off of ΔSUV_max_ could predict both pCR and prognosis in TNBC patients (10, 14). Generally, in triple-negative breast cancer patients, pCR to NAC is associated with improved prognosis. However, it has been reported that the pCR did not always affect disease-free or overall survival in triple-negative breast cancer (15). Furthermore, Groheux et al. reported that the clinical relevance of PET for everyday practice is still limited, and the findings cannot be used outside clinical trials (10). Moreover, the devices and the methods of PET are not the same among institutions. Therefore, the results cannot be simply compared with those of other institutions, and a standard value for every institution is needed.

In some previous studies, PET data acquisition started at 60 min after injection (10, 14, 18). However, most normal tissues have decreased background activity, and most malignant lesions have increased ^18^F-FDG uptake on delayed time-point images, leading to higher lesion-to-background ratios and, thus, higher sensitivity (19). Therefore, in this study, PET data acquisition started at 90 min after injection. However, the results of previous studies and those of the present study cannot be directly compared.

Pre-treatment SUV must be high to detect a meaningful reduction during treatment. Triple-negative breast cancers are known to be aggressive and have high FDG uptake (16, 17, 18). In the present series, only 1 (3.1%) tumor had SUV_max_ <3 at baseline. There was a significant correlation between high FDG uptake after NAC and non-pCR in TNBC patients (Table 2). However, because of the aforementioned reason, the results of previous studies and those of the present study could not be directly compared.

In summary, the change in ^18^F-FDG tumor uptake after NAC offers effective stratification of TNBC patient outcomes. It identifies poor metabolic responders in whom the planned NAC regimen could result in non-pCR tumor and a high risk of early relapse. Thus, FDG-PET/CT at baseline and after NAC should be useful for patient selection to recommend closer follow-up after surgery to detect relapse early.

## Conclusion

This study showed that FDG-PET/CT at baseline and after NAC could predict the pathological response to NAC before surgery and the clinical outcome after surgery in locally advanced TNBC patients. Patients who do not achieve pCR and poor responders are at high risk of early relapse, and closer follow-up is necessary in these patients to detect relapse early.

## References

[1] Foulkes WD, Smith IE, Reis-Filho JS (2010). Triple-negative breast cancer. N Engl J Med.

[2] Boyle P (2012). Triple-negative breast cancer: epidemiological considerations and recommendations. Ann Oncol.

[3] Liedtke C, Mazouni C, Hess KR, André F, Tordai A, Mejia JA (2012). Response to neoadjuvant therapy and long-term survival in patients with triple-negative breast cancer. J Clin Oncol.

[4] Von Minckwitz G, Untch M, Blohmer JU, Costa SD, Eidtmann H, Fasching PA (2012). Definition and impact of pathologic complete response on prognosis after neoadjuvant chemotherapy in various intrinsic breast cancer subtypes. J Clin Oncol.

[5] Fuster D, Duch J, Paredes P, Velasco M, Muñoz M, Santamaría G (2008). Preoperative staging of large primary breast cancer with [18F]fluorodeoxyglucose positron emission tomography/computed tomog-raphy compared with conventional imaging procedures. J Clin Oncol.

[6] Groheux D, Hindié E, Delord M, Giacchetti S, Hamy AS, de Bazelaire C (2012). Prognostic impact of (18)FDG-PET-CT findings in clinical stage III and IIB breast cancer. J Natl Cancer Inst.

[7] Schwarz-Dose J, Untch M, Tiling R, Sassen S, Mahner S, Kahlert S (2009). Monitoring primary systemic therapy of large and locally advanced breast cancer by using sequential positron emission tomography imaging with [18F] fluorodeoxyglucose. J Clin Oncol.

[8] Groheux D, Giacchetti S, Espié M, Rubello D, Moretti JL, Hindié E (2011). Early monitoring of response to neoadjuvant chemotherapy in breast cancer with 18F-FDG PET/CT: defining a clinical aim. Eur J Nucl Med Mol Imaging.

[9] Nakajima N, Sugawara Y, Kataoka M, Hamamoto Y, Ochi T, Sakai S (2013). Differentiation of tumor recurrence from radiation-induced pulmonary fibrosis after stereotactic ablative radiotherapy for lung cancer: characterization of 18F-FDG PET/CT findings. Ann Nucl Med.

[10] Groheux D, Hindié E, Giacchetti S, Hamy AS, Berger F, Merlet P (2014). Early assessment with ^18^F-fluorodeoxyglucose positron emission tomography/computed tomography can help predict the outcome of neoadjuvant chemotherapy in triple negative breast cancer. Eur J Cancer.

[11] Nagao T, Kinoshita T, Hojo T, Tsuda H, Tamura K, Fujiwara Y (2012). The differences in the histological types of breast cancer and the response to neoadjuvant chemotherapy: the relationship between the outcome and the clinicopathological characteristics. Breast.

[12] Cortazar P, Zhang L, Untch M, Mehta K, Costantino JP, Wolmark N (2014). Pathological complete response and long-term clinical benefit in breast cancer: the CTNeoBC pooled analysis. Lancet.

[13] Groheux D, Giacchetti S, Delord M, de Roquancourt A, Merlet P, Hamy AS (2015). Prognostic impact of 18F-FDG PET/CT staging and of pathological response to neoadjuvant chemotherapy in triple-negative breast cancer. Eur J Nucl Med Mol Imaging.

[14] Groheux D, Hindié E, Giacchetti S, Delord M, Hamy AS, de Roquancourt A (2012). Triple-negative breast cancer: early assessment with 18F-FDG PET/CT during neoadjuvant chemotherapy identifies patients who are unlikely to achieve a pathologic complete response and are at a high risk of early relapse. J Nucl Med.

[15] Von Minckwitz G, Loibl S, Untch M, Eidtmann H, Rezai M, Fasching PA (2014). Survival after neoadjuvant chemotherapy with or without bevacizumab or everolimus for HER2-negative primary breast cancer (GBG 44-GeparQuinto). Ann Oncol.

[16] Groheux D, Espié M, Giacchetti S, Hindié E (2013). Performance of FDG PET/CT in the clinical management of breast cancer. Radiology.

[17] Groheux D, Giacchetti S, Moretti JL, Porcher R, Espié M, Lehmann-Che J (2011). Correlation of high 18F-FDG uptake to clinical, pathological and biological prognostic factors in breast cancer. Eur J Nucl Med Mol Imaging.

[18] Groheux D, Majdoub M, Sanna A, de Cremoux P, Hindié E, Giacchetti S (2015). Early metabolic response to neoadjuvant treatment: FDG PET/CT criteria according to breast cancer subtype. Radiology.

[19] Cheng G, Torigian DA, Zhuang H, Alavi A (2013). When should we recommend use of dual time-point and delayed time-point imaging techniques in FDG PET?. Eur J Med Mol Imaging.

